# Connectomic signatures advance the understandings of transdiagnostic heterogeneity in psychosis

**DOI:** 10.1038/s43856-026-01967-y

**Published:** 2026-10-10

**Authors:** Yu Du, Jingjing Zhang, Ting Ma, Chenfei Ye

**Affiliations:** 1 School of Information Science and Technology, Harbin Institute of Technology (Shenzhen), Shenzhen, China; 2 Department of Psychiatry, Ruijin Hospital, Shanghai Jiao Tong University School of Medicine, Shanghai, China; 3 School of Biomedical Engineering, Harbin Institute of Technology (Shenzhen), Shenzhen, China

**Keywords:** Psychiatric disorders, Functional magnetic resonance imaging

## Abstract

Current consensus-driven diagnostic systems poorly capture the marked heterogeneity and high comorbidity of psychiatric disorders, necessitating brain-based transdiagnostic frameworks to advance mechanistic understanding and clinical translation. Connectome-wide association studies have consistently identified widespread large-scale dysconnectivity across diagnostic boundaries, yet normative modeling and meta-analytic evidence highlights substantial inter-individual variability in the spatial topography and magnitude of connectomic deviations. In this Perspective, we bridge this divide between cross-disorder commonalities and individualized pathology by integrating connectomic overlap and computational biotyping into a top-down, four-tiered hierarchical architecture. This framework progresses from macro-scale transdiagnostic topological scaffolds, through meso-scale phenotypic triangulation, to data-driven biotypes and micro-scale clinical translations, delineating bidirectional interplay between shared network constraints and heterogeneous pathological diversification. Building on this architecture, we outline strategies leveraging multi-cohort data harmonization and healthy-anchored normative biotyping to refine robust cross-disorder circuits and reproducible neurobiotypes. Lastly, we propose a translational roadmap to transition connectomic stratification toward prospective, treatment-relevant clinical validation in precision psychiatry.

## Introduction

Current clinical neuroscience research predominantly relies on consensus-driven diagnostic criteria to characterize psychiatric disorders, such as the Diagnostic and Statistical Manual of Mental Disorders, Fifth Edition (DSM-5)^1^, and the International Classification of Diseases, Tenth Revision (ICD-10)^2^. These criteria mainly depend on patient self-reports and clinician evaluations of behavior, rather than on the identification of neuropathological anomalies, in part due to the complex and elusive nature of psychiatric disorders^3^. Over the past decade, an increasing body of research has indicated that the existing consensus-based diagnostic criteria do not adequately capture the diversity and high rates of comorbidity observed in psychiatric disorders^4,5^. This limitation likely contributes to an incomplete understanding of psychopathology and to the suboptimal clinical efficacy of available treatments.

To overcome the constraints imposed by the traditional case–control diagnostic model, many recent studies have endeavored to establish novel, dimensional frameworks for characterizing psychiatric disorders, in line with the Research Domain Criteria (RDoC) framework^6^. In this context, the transdiagnostic concept has emerged as a promising direction in psychiatry. Rather than treating diagnostic categories as mutually exclusive entities, the transdiagnostic approach aims to identify mechanisms and dimensions that cut across multiple disorders, thereby offering a more nuanced classification system that goes beyond categorical boundaries.

Deviations in brain connectivity patterns are evident across all major mental disorders, indicating that disruptions in large-scale connectomic organization and interregional coordination are central to a variety of psychological dysfunctions. The growing recognition of connectome reconfiguration as a pivotal element in psychopathology has been bolstered by recent findings from connectome-wide association studies (CWAS)^7,8^. CWAS systematically examine associations between whole-brain connectivity profiles and clinical or behavioral phenotypes, and thus are poised to unlock new insights into the neural underpinnings of psychopathology across the entire brain^9–12^. These studies are crucial for delineating the reconfiguration of brain-network topologies in relation to the diverse manifestations of mental symptoms. However, despite substantial progress, key questions remain unresolved. On the one hand, normative modeling and large-scale meta-analytic work have highlighted substantial heterogeneity in brain structural and functional deviations in individuals with psychosis, even within the same diagnostic category^13–17^. On the other hand, cross-disorder imaging studies and theoretical models suggest the existence of shared connectomic substrates of psychiatric risk^18,19^. Reconciling these apparently divergent observations—strong individual differences and robust transdiagnostic overlap—is essential for advancing a mechanistic understanding of psychosis.

**Fig. 1:**
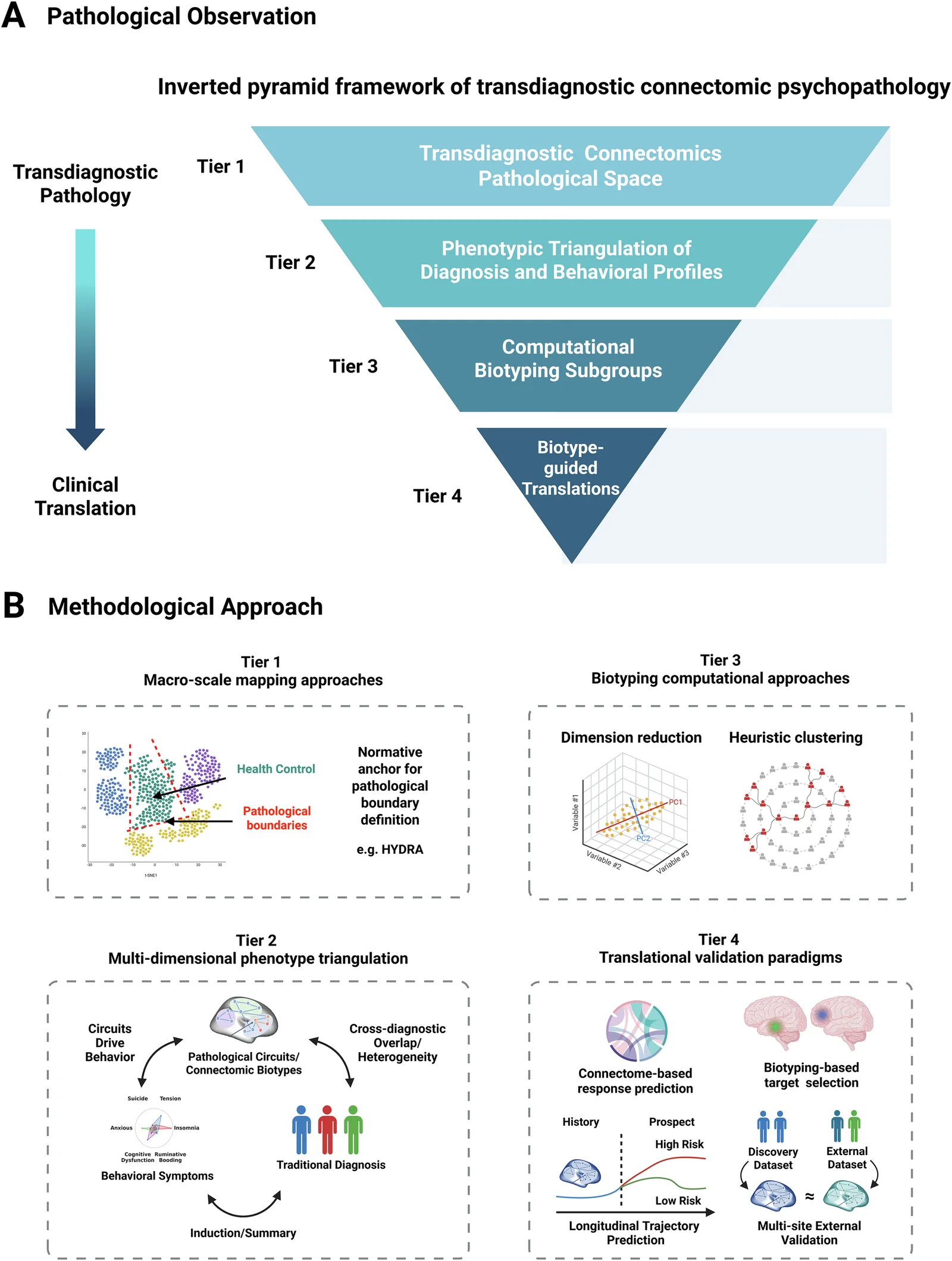
A four-tiered inverted pyramid framework for transdiagnostic connectomic psychopathology. **A** Schematic overview of the top-down pathological observation hierarchy. The framework systematically progresses from the macro-scale transdiagnostic connectomic pathological space (Tier 1), through meso-scale phenotypic triangulation of diagnostic categories and behavioral profiles (Tier 2) and data-driven computational biotyping subgroups (Tier 3), down to the micro-scale biotype-guided clinical translation (Tier 4), delineating the gradient from shared pathological features to individualized clinical manifestations. **B** Corresponding methodological approaches for each hierarchical level. Each tier matches the phenomenological level in (**A**), summarizing core analytical approaches and experimental paradigms used to characterize the corresponding connectomic features, from macro-scale whole-brain mapping to clinical translational validation.

Against this backdrop, the present article synthesizes current evidence to reconceptualize the relationship between connectomic overlap and biotyping in the study of transdiagnostic heterogeneity in psychosis. Rather than treating cross-disorder commonalities and individualized subtyping as separate lines of inquiry, we integrate them into a mechanistically progressive “inverted pyramid” hierarchical framework that systematically organizes findings from macro-scale topological commonalities down to micro-scale individual residuals (see Fig. 1). Within this framework, we delineate how heterogeneous pathological changes are constrained by large-scale network architecture, and how multi-level connectomic signatures collectively contribute to the phenotypic landscape of psychosis. Building on this integrated perspective, we then offer specific practical recommendations regarding data integration strategies and healthy-anchored computational biotyping approaches, and outline a translational roadmap toward clinically actionable connectomic stratification in precision psychiatry.

## A four-tiered inverted pyramid framework for transdiagnostic connectomics

Current connectomic research in psychopathology has long treated transdiagnostic comorbidity mechanisms and multi-dimensional connectomic alterations within individual disorders in isolation. In fact, connectomic overlapping and individualized biotyping are by no means mutually exclusive; instead, they can be integrated into a top-down, mechanistically progressive “inverted pyramid” hierarchical framework (see Fig. 1). By converging systematically from macro-scale topological commonalities to micro-scale individualized clinical translations, this architecture addresses how heterogeneous pathology is constrained by large-scale network organization while offering a systematic methodological roadmap for precision psychiatry.

This framework is organized along two complementary dimensions: a phenomenological hierarchy of connectomic pathology from macro to micro scales (Fig. 1A), and a corresponding methodological toolkit of analytical approaches for each level (Fig. 1B). Together, they provide an integrated view of both biological observations and the methods used to delineate them.

### Macro-scale transdiagnostic pathological scaffold

At the macro-scale pinnacle of this pyramid, diverse psychiatric conditions (e.g., the transdiagnostic psychosis spectrum) share highly overlapping connectomic disturbances within the whole-brain topological space. The macro-scale architecture of the human connectome fundamentally constrains the ways in which structural and functional alterations manifest across diagnostic boundaries. Early conceptual work highlighted the human connectome as a common substrate for transdiagnostic risk^18^, and subsequent empirical evidence has demonstrated that macro-scale network organization shapes disorder-related alterations. For instance, a multi-site cross-disorder study by de Lange and colleagues^20^ spanning eight psychiatric and neurological conditions revealed that rich-club connections—long-range, high-capacity pathways critical for global communication and network integration—exhibit pronounced vulnerability across diverse disorders. These findings support the notion that transdiagnostic susceptibility is partly governed by connectome architecture, with core hubs and integrative systems serving as common points of failure.

Traditional cross-disorder mapping approaches predominantly rely on simplistic linear models, which fail to capture the high-order and non-linear properties of transdiagnostic connectome reorganization. To overcome this bottleneck, advanced deep-learning frameworks, such as the multivariate distance-based connectome network (MDCN)^11^, have been developed to model complex dysconnectivity patterns. By pooling large-scale resting-state fMRI datasets, MDCN revealed substantial regional overlaps in functional network disturbances—particularly within the left visual, left salience/ventral attention, and bilateral default mode networks—thereby providing non-linear neurobiological validation for shared substrates between clinically distinct profiles like ASD and ADHD.

Notably, although network alterations across diverse psychiatric and neurological disorders frequently converge on core hub nodes within whole-brain topology, such a “single-axis” neuroalteration model fails to account for the substantial diversity in clinical phenotypes. In practice, the shared connectomic scaffold provides a foundation for identifying generalizable therapeutic targets, whereas disease-specific regions define the phenotypic boundaries of distinct conditions. These specific regions enable the delineation of intra-disorder subtypes and distinct pathological mechanisms, thereby informing precision medicine and individualized treatment strategies. Utilizing multimodal biomarkers—such as neuroimaging^21^ and speech features^22^ —allows for the identification of disease-specific biological fingerprints, enhancing accurate multi-class differential diagnosis and reducing misclassification. Consequently, when defining the pathological space, concurrently capturing connectomic overlap and heterogeneity (biotyping) is crucial for understanding and translating transdiagnostic connectomic features.

### Meso-scale phenotypic triangulation across diagnoses and behavior

The second tier incorporates consensus-based traditional clinical diagnoses. Although the mutually exclusive nature of current diagnostic categories has been widely criticized for ignoring the spectrum-like and comorbid traits of psychiatric disorders, traditional diagnostic systems remain a useful reference frame bridging biology and behavior in transdiagnostic connectomics research.

At this level, a critical open question is whether shared connectomic signatures are sufficient to predict complex behavioral phenotypes in a transdiagnostic fashion. Compelling phenotypic validation was provided by Tong et al.^23^, who utilized connectome-based predictive modeling (CPM) in a large transdiagnostic cohort. The authors demonstrated that whole-brain functional connectivity could reliably predict individual-level cognitive capacity (Full-Scale Intelligence Quotient, FSIQ) across the traditional boundaries of ASD, MDD, ADHD, and anxiety disorders, exhibiting robust generalizability across independent replication cohorts.

Accordingly, we emphasize that the primary objective at this tier should shift from the simplistic validation of shared or unique circuits to a granular mapping onto specific symptom dimensions and cognitive-behavioral phenotypes. A framework of “phenotypic triangulation” among pathological circuits, traditional diagnoses, and behavioral symptoms is thus established. Through this multi-dimensional mapping, researchers can test two complementary mechanistic hypotheses. First, whether transdiagnostic circuits overlap directly underlie generalized symptoms that cut across diagnostic boundaries. For instance, Goldstein-Piekarski et al.^24^ demonstrated that shared network circuit dysfunctions in depression and anxiety disorders mediate transdiagnostic symptom dimensions common to both conditions. Second, whether disorder-specific circuit anomalies account for core behavioral phenotypes unique to traditional diagnostic categories.

### Computational biotyping: data-driven neurobiological subgroups

The third tier comprises computational biotypes that emerge within the shared transdiagnostic pathological space due to distinct circuit deviation trajectories. Rather than grouping patients solely by clinical labels, this data-driven clustering approach seeks to delineate subgroups characterized by shared neurobiological mechanisms, thereby providing mechanical explanations for the co-occurrence and heterogeneity of clinical manifestations^25^.

To date, a diverse methodological ecosystem for connectomic biotyping has evolved. Hierarchical and k-means clustering remain the most widely adopted unsupervised techniques due to their conceptual simplicity and robust performance in major depression^25,26^ and the schizophrenia-bipolar spectrum^27^. Beyond these classical algorithms, non-parametric techniques such as multiple co-clustering have been designed to isolate distinct neurophysiological subtypes of depression^28^, while heuristic community detection algorithms (e.g., the Walktrap method) have successfully recovered directed and undirected connectivity paths to reveal biotypes with differential clinical profiles^29^. To enhance computational efficiency and cluster separability in high-dimensional spaces, recent workflows have integrated dimensionality reduction with canonical clustering, employing spectral clustering^30^, kernel-based clustering^31^, and non-negative matrix factorization^32^ to successfully discriminate biotypes in first-episode antipsychotic-naïve schizophrenia, ASD, and ADHD. Concurrently, deep learning-based biotyping has gained traction; for example, applying deep stacked autoencoders combined with ensemble clustering revealed two distinct biotypes within a broad psychotic-affective spectrum that differed in frontal-posterior functional balance^33^. Similarly, self-supervised learning frameworks like Explainability-Augmented Region of Interest Selection (EAG-RS) have been introduced to capture high-order functional associations, identifying three distinct ASD biotypes with differential symptom profiles^34^.

Collectively, these methodological advances illustrate a broader trend toward increasingly sophisticated, high-dimensional biotyping pipelines capable of resolving finer-grained neurobiological subgroups. As algorithmic complexity increases, however, so does the importance of ensuring that derived biotypes correspond to meaningful clinical distinctions rather than reflecting idiosyncrasies of particular datasets or analytical choices—a consideration that becomes particularly salient when evaluating the translational potential of connectomic subtyping.

### Biotype-guided translations

At the final foundation of the inverted pyramid lies biotype-guided translations—the idiosyncratic connectomic fluctuations unique to each patient that cannot be fully captured by mid-level biotypes. As normative modeling research in psychotic disorders has revealed, even though patients as a cohort frequently exhibit extreme deviations exceeding healthy baselines, the precise spatial pattern, temporal dynamics, and magnitude of these deviations remain highly heterogeneous across individuals, persisting even within identical clinical categories. Once the macro-scale transdiagnostic space, mid-scale diagnostic coordinates, and mid-to-micro-scale biotype features are sequentially filtered, the remaining core residual variance represents the ultimate frontier of precision psychiatry.

Translating these connectomic insights into clinical therapeutics, however, remains an ongoing endeavor. At present, connectomics-guided interventions are primarily in an exploratory and preliminary validation phase. Although a growing body of literature demonstrates its potential across diverse applications^24,35^—including adjunctive diagnosis, treatment response forecasting, target localization^36^, and neurosurgical planning across neuropsychiatric^37^ and consciousness disorders^38^—the current empirical evidence remains constrained. Most available studies rely on observational designs or unblinded pilot trials, lacking support from large-sample, multi-center, double-blind randomized controlled trials (RCTs)^38^. Furthermore, high heterogeneity and susceptibility^39^ to imaging artifacts mean that current brain-based biomarkers alone are insufficient to independently dictate standalone therapeutic decisions.

To address these translational bottlenecks without abandoning brain-centric mechanisms, this tier of clinical translations supports a highly feasible dual-stage paradigm. This paradigm is characterized by mid-scale qualitative guidance (where computational biotypes inform intervention mechanisms) paired with micro-scale locational refinement (where individual residual connectomic profiles inform precise spatial targets). To operationalize this framework, expanding sample sizes through unified multi-center data aggregation and implementing prospective predictive designs alongside operational validation trials represent viable paths forward. Anchoring these methods within a healthy reference framework and resolving the phenotypic triangle turns patient-to-patient noise into actionable targets for precision therapy.

## Implications and future directions

Given the varying engagement of dysconnectivity across psychiatric disorders, each illness and each individual may be better conceptualized as occupying a position between transdiagnostically shared patterns and disorder-specific or person-specific patterns of connectome disruption. Within this framework, connectomic overlapping and connectomic biotyping are intrinsically linked: overlap characterizes the shared network-level constraints that shape where alterations preferentially emerge across disorders (e.g., hub- and rich-club–related vulnerability and canonical systems such as default mode and salience/attention networks), whereas biotyping explains how individuals diverge from these shared constraints through distinct deviation configurations. Mechanistically, overlap can be treated as a “common scaffold” that defines general liability and yields robust, generalizable signatures, while biotyping partitions the residual variance around this scaffold into subgroups that may reflect different pathophysiological routes to similar symptom syndromes. Importantly, the relationship is bidirectional: overlap-informed priors can stabilize biotyping solutions and reduce spurious clustering driven by sampling, preprocessing, or site effects; conversely, reproducible biotypes can clarify whether an apparent overlap is truly universal or is dominated by particular subgroups. Integrating these two levels therefore provides a coherent route to reconceptualize psychosis nosology in circuit terms and to move from descriptive group differences toward clinically actionable stratification in precision psychiatry. Building on this integrated perspective, we outline three interlinked priorities to advance transdiagnostic connectomic translation, addressing data standardization, pathological space definition, and clinical validity in sequence.

### Data integration and harmonization

**Fig. 2:**
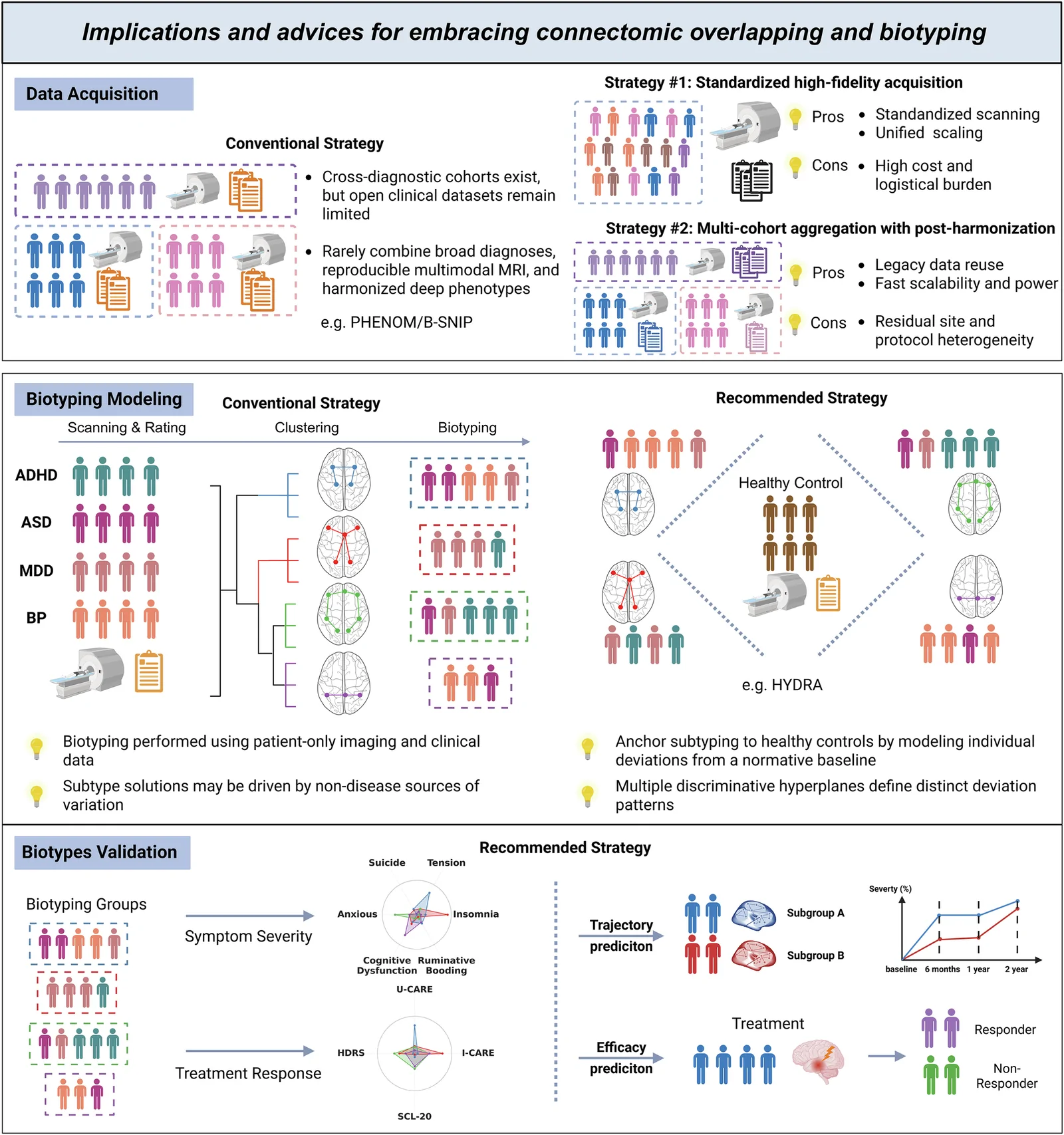
Actionable methodological roadmap for implementing connectomic overlap and biotyping in transdiagnostic psychosis research. Top: Data acquisition—contrasts the limitations of conventional cross-diagnostic cohort designs with two complementary optimized strategies—standardized harmonized acquisition and multi-cohort retrospective harmonization—and their respective strengths and trade-offs. Middle: Biotyping modeling—compares conventional patient-only unsupervised clustering, which is vulnerable to non-disease confounding variation, with a recommended healthy-anchored normative biotyping approach exemplified by the HYDRA framework. Bottom: Biotype validation—illustrates the recommended paradigm shift from internal clustering metrics to prospective, clinically relevant external validation, including symptom trajectory and treatment efficacy prediction, to facilitate clinical translation. HYDRA Heterogeneity through Discriminative Analysis.

A major bottleneck is data availability and standardization (see the top panel in Fig. 2). Although cross-diagnostic cohorts such as B-SNIP^40^ and PHENOM^41^ exist, conventional data acquisition requires caution. This challenge is compounded by two key issues: a scarcity of openly shared, clinically focused datasets, and a lack of resources that simultaneously provide wide diagnostic coverage, reproducible multimodal MRI, and harmonized deep phenotyping—all essential for robust brain-behavior mapping and prospective validation. This critical gap can be bridged either by initiating newly established datasets or by aggregating existing multi-center cohorts.

For newly initiated data collection, to maximize the interpretability of transdiagnostic comparisons, researchers must ensure that data acquisition operates within a unified framework by adopting a standardized, “HCP-like” acquisition protocol. A premier example is the Transdiagnostic Connectome Project (TCP)^42^, which utilizes harmonized protocols and workflows to maximize measurement precision and cross-site comparability while reinforcing multimodal data integration. This standardized data infrastructure enables reliable fine-grained characterization of transdiagnostic dimensional symptom profiles that transcend traditional diagnostic boundaries. Leveraging this cohort, recent work demonstrates that brain-network dynamics are flattened across cognitive states in individuals with psychiatric illness and can robustly delineate internalizing, externalizing, cognitive, and social/reward symptom dimensions with greater discriminative power than both binary case–control status and primary diagnostic grouping. This design enables a more precise estimation of effect sizes, and its comprehensive clinical assessments provide critical data support for exploring the symptomatic homogeneity and heterogeneity driven by connectomic overlap and biotyping. Although this design requires substantial resource investment, its high degree of standardization establishes a solid foundation for constructing reference-grade datasets and benchmarking analytical workflows.

Conversely, for studies repurposing legacy multi-cohort data, researchers must implement rigorous harmonization techniques to enhance cross-cohort comparability. A case in point is the Reproducible Brain Charts (RBC)^43^ initiative, which aggregates existing datasets under a unified, reproducible preprocessing pipeline and consistent phenotypic definitions. This strategy rapidly achieves large sample sizes, broad diagnostic coverage, and robust population representation, which are exceptionally valuable for detecting shared features, quantifying heterogeneity, and enhancing out-of-sample generalizability. Last but not least, both TCP and RBC exemplify a key best practice: releasing data in the Brain Imaging Data Structure (BIDS) format on open platforms like OpenNeuro, thereby substantially facilitating data reuse, result replication, and methodological development.

### Delineating the pathological space with a healthy anchor

Given the high clinical heterogeneity and inherent ascertainment biases within patient cohorts, defining the absolute boundaries of the pathological space solely within clinical samples remains challenging and error-prone. To address this limitation, integrating large-scale healthy control datasets is essential for establishing a normative anchor. By mapping the baseline topological distribution of the healthy brain, any individual deviation exceeding normal statistical variation can be precisely mapped as an individualized pathological space. Methodologically, given the strong age dependence and developmental modulation of connectomic features, we recommend that future research on pathological overlap and biotyping more explicitly incorporate the healthy brain as an “anchor” (see the middle panel in Fig. 2).

From this perspective, normative modeling and hierarchical Bayesian approaches^44,45^ are gaining increasing recognition. These methods quantify individualized deviations relative to age- and site-aware normative reference models, providing a more precise characterization than traditional case–control contrasts. As feature extractors for evaluating deviations from a normal baseline, these approaches can construct an individual “disease deviation map”, providing a critical foundation for further transdiagnostic analyses of pathological overlap and biotyping.

Concurrently, from the dimension of feature representation, contrastive learning-based methods—such as contrastive principal component analysis^46^ (cPCA)—offer an alternative strategy for isolating background noise. By leveraging connectomic differences between clinical and healthy populations, cPCA identifies low-dimensional projections that maximize disease-unique variance. Unlike conventional PCA, which is susceptible to interference from pathology-irrelevant background variables (e.g., age, sex, and batch effects), cPCA suppresses shared background noise to extract disease-specific signals masked by traditional approaches. This continuous projection not only captures shared disease variance but also identifies diagnosis- or symptom-specific pathological features.

This anchor-based perspective is equally relevant for biotyping: models can cluster these “deviation profiles”—i.e., how individuals depart from a normative reference across distributed circuits and networks—rather than raw patient connectomes, as the latter easily causes clusters to reflect irrelevant confounding structures. The Heterogeneity through Discriminative Analysis (HYDRA) framework^47–49^ provides a clear discriminative template for such discrete subtyping: by utilizing the healthy control distribution as an enclosing anchor, it employs multiple hyperplanes to identify patient subgroups that deviate from this anchor in distinct patterns. Consequently, it directly links subtyping to unique modes of pathological deviation rather than arbitrary similarities in high-dimensional feature spaces. Extending this logic to multimodal connectomics and multilevel representations that integrate edges, circuits, and canonical networks will be essential for testing whether network-level overlaps quantitatively track clinical similarity across both diagnoses and data-driven biotypes.

### Moving beyond internal metrics: prospective external validation of biotypes

Accumulating empirical evidence has begun to demonstrate the clinical utility of connectomic signatures for prognostic and predictive stratification across transdiagnostic psychiatric spectra. For psychosis spectrum conditions, Sarpal et al.^50^ established a resting-state fMRI-based striatal connectivity index that predicted antipsychotic response with an AUC = 0.78 in both first-episode schizophrenia and independent chronic psychosis cohorts, and further correlated with hospital length of stay. Extending beyond single-region seeds to whole-brain signatures, Cao et al.^51^ identified a paradigm-independent connectomic biomarker for individualized antipsychotic response in first-episode psychosis, with robust performance replicated in both discovery and independent validation samples. Beyond psychosis, Zhang et al.^52^ validated transdiagnostic generalizability in internalizing psychopathologies, showing that whole-brain functional connectivity predicted multidimensional symptom improvement across six diagnostic categories of depression and anxiety, and generalized across cognitive-behavioral therapy, pharmacotherapy, and supportive therapy modalities. Collectively, these findings span focal circuit markers to distributed connectome-wide signatures, cover both pharmacological and psychosocial interventions, and provide preliminary proof-of-concept for connectomics-guided clinical decision-making.

Despite these promising findings, the clinical translation of connectomic biotyping remains at an early stage, constrained by notable evidence gaps and pervasive methodological limitations. Most available findings are derived from retrospective or single-site studies, and large-scale, multi-center prospective validation remains a critical barrier to routine clinical implementation. More broadly, Winter and Hahn^53^ have highlighted a systemic methodological flaw across the field: the majority of biotyping studies—including most deep learning-based connectomic subtyping work—fail to rigorously evaluate the statistical significance, stability, and out-of-sample generalizability of their derived subtypes. Instead, validity is frequently inferred solely from internal clustering performance, which is susceptible to arbitrary analytical choices and dataset-specific idiosyncrasies rather than reflecting clinically meaningful neurobiological heterogeneity.

To address this replication gap and strengthen clinical translatability, the evaluation of connectomic biotypes must shift from a primary focus on internal clustering metrics to prospective, outcome-relevant external validation (see the bottom panel in Fig. 2). Divergent analytical pipelines—varying in feature definition, dimensionality reduction, distance metrics, and hyperparameters—can yield divergent “biotype” solutions even from the same dataset. For this reason, biotype validity cannot be judged primarily by within-cluster versus between-cluster indices or apparent stability under resampling. The decisive criterion is clinical utility: whether derived subtypes can prospectively predict clinical outcomes or differential treatment response beyond what is explained by conventional diagnostic labels and baseline symptom severity.

Two complementary, clinically oriented validation designs are particularly well suited to this goal. The first is pathology trajectory prediction, which forecasts the longitudinal evolution of symptoms and functional outcomes based on baseline connectomic profiles. In this paradigm, biotypes identified at study entry are tested for their ability to predict subsequent changes in symptom severity, illness course, and functional decline over months to years, independent of baseline diagnosis and clinical severity. This design directly assesses whether connectomic stratification captures prognostically meaningful neurobiological heterogeneity rather than transient cross-sectional differences, and can be implemented in naturalistic prospective cohorts without uniform treatment assignment. For example, Li et al.^54^ predicted baseline progression rate phenotypes in ALS from DTI white matter structural connectivity using linear classifiers, whereas Maheux et al.^55^ forecasted 1.5–4.5-year cognitive trajectories in Alzheimer’s disease from multimodal baseline data, including hippocampal volumetry. The second is efficacy prediction, which examines whether baseline connectomic biotypes forecast differential response to specific interventions. Under standardized trial conditions, patients receive uniform treatments, and predictive validity is established by observing significantly divergent symptom improvement and connectomic remediation trajectories across subgroups. Early evidence in depression has demonstrated this principle by identifying connectivity-defined subtypes that predict responsiveness to repetitive transcranial magnetic stimulation^26^, and a recent large-scale study across multiple depression and anxiety cohorts confirmed that individualized circuit metrics can predict differential benefit from pharmacotherapy and behavioral therapy at scale^56^.

For transdiagnostic psychosis research, analogous validation efforts should prioritize prospective designs, harmonized intervention protocols, and preregistered endpoints. The goal is to demonstrate that connectome-derived subgroups generalize across sites and meaningfully stratify prognosis and treatment response. In this way, connectomic biotyping can advance from plausible substructure discovery to a clinically testable framework, and transdiagnostic connectomic overlap can be operationalized not only as a shared risk substrate but also as a set of constraints that guide robust, treatment-relevant stratification.

## Supplementary information

Transparent Peer Review file
