# Supplementary material for: Connectomic signatures advance the understandings of transdiagnostic heterogeneity in psychosis
Source: Commun Med (Lond). 2026 Oct 10;6:526. doi: 10.1038/s43856-026-01967-y (PMC13653919; doi:10.1038/s43856-026-01967-y)
Supplement: Supplementary file 1 [file 43856_2026_1967_MOESM1_ESM.pdf]

nature portfolio

Peer Review File

Connectomic signatures advance the understandings of transdiagnostic heterogeneity in psychosis

Corresponding Author: Dr Chenfei Ye

This file contains all reviewer reports in order by version, followed by all author rebuttals in order by version.

Version 0:

Reviewer comments:

Reviewer #1

(Remarks to the Author)
This manuscript argues that connectomic signatures can advance our understanding of transdiagnostic heterogeneity within psychosis. By reviewing relevant literatures, the authors aim to bridge studies showing connectome-wide associations within and between disorders with those establishing substantial between-person heterogeneity. They conclude with several practical recommendations and directions for the field. Overall this is a nice, concise and timely contribution that will help researchers think about where the field should go. I have a few comments that may improve the manuscript.

I appreciated the conceptual focus on different levels of phenotypic analysis, from individuals to diagnostic subgroups to diagnoses overall to transdiagnostic classes. This could be fleshed out more, however, as the connections between these layers seems to be the essence of the problem the authors want to solve. It was not always clear whether the authors think the proposed methodologies are uniquely suited to characterize one level of that hierarchy better than others, if one level is more important than others, or if they are simply intrinsically connected and should always be addressed simultaneously.

The authors note that the decisive criterion for validating biotypes is clinical utility. This is true, and the paper should provide a better description of where the field stands in terms of clinically useful connectomic signatures/biotypes/etc. Where are we now with actually using connectomics to inform treatment? How numerous, diverse, and strong are the studies? The authors note one individual study and one meta-analysis in line 274, but given that this is the primary objective of this work, stated a few times in the manuscript, context for where the field stands is important. This is especially the case given concerns that brain-level metrics, especially with available technology, may be insufficient for making real treatment decisions.

The recommendations are strong and appreciated.

Reviewer #2

(Remarks to the Author)
Dear authors, thanks for sharing with us this manuscript. Here is my review.

The central problem with this manuscript is that its contribution relative to the existing literature is unclear. The transdiagnostic connectomics perspective has been articulated in prior work, including Buckholtz and Meyer-Lindenberg (2012), which the authors cite as reference [18], and Vanes and Dolan (2021), cited as reference [19]. The conceptual distinction between shared network-level constraints (overlapping) and individual deviation patterns (biotyping) is a useful organizational device, but it does not constitute a novel theoretical advance. The recommendations in the final section, namely standardized data acquisition, normative anchoring, and prospective validation, reflect established best practices in the field rather than new proposals.

The scope of the literature covered is also relatively narrow for a review paper. A narrative, non-systematic and non-quantitative review can be valuable when it synthesizes a broad literature into a new interpretive framework or when it identifies non-obvious gaps, but the present manuscript does not attempt comprehensive coverage, does not perform any quantitative synthesis, and does not propose a genuinely novel conceptual model.

I would encourage the authors to consider what specific question or gap motivated this review and to restructure the manuscript around that contribution, whether it takes the form of a systematic synthesis, a novel theoretical framework, or an original analysis. The topic itself is timely and clinically important, and there is room for a review that makes a distinctive contribution to the transdiagnostic connectomics literature.

Version 1:

Reviewer comments:

Reviewer #1

(Remarks to the Author)
The authors have done a nice job of addressing my prior comments. I think the paper is improved.

The new figures are especially interesting. I do have one constructive comment about the inverted pyramid framework though. It seems to combine biological/psychopathological levels of observation with the methodologies used to identify them. For the figure to be most helpful, these should probably be separated if both are intended to be included. At a minimum, it should be explicitly acknowledged that structures and methods are reflected, and probably each level of the hierarchy should display both levels of observation and methods (if both are deemed equally relevant by the authors).

Reviewer #2

(Remarks to the Author)
Dear authors,

thanks for addressing my (our) revisions. I don't have any further objection towards pubblication.

Version 2:

Reviewer comments:

Reviewer #1

(Remarks to the Author)
The authors have addressed my comment regarding the inverted pyramid figure, which I think is improved from prior versions. I have no further comments.

Open Access This Peer Review File is licensed under a Creative Commons Attribution 4.0 International License, which permits use, sharing, adaptation, distribution and reproduction in any medium or format, as long as you give appropriate credit to the original author(s) and the source, provide a link to the Creative Commons license, and indicate if changes were made.
In cases where reviewers are anonymous, credit should be given to 'Anonymous Referee' and the source.

The images or other third party material in this Peer Review File are included in the article's Creative Commons license, unless indicated otherwise in a credit line to the material. If material is not included in the article's Creative Commons license and your intended use is not permitted by statutory regulation or exceeds the permitted use, you will need to obtain permission directly from the copyright holder.
To view a copy of this license, visit https://creativecommons.org/licenses/by/4.0/

Responses to reviewers

We sincerely thank both reviewers for their thoughtful and constructive comments, which have greatly helped us refine and strengthen the manuscript. Our detailed responses to each comment are provided below.

Reviewer: 1

This manuscript argues that connectomic signatures can advance our understanding of transdiagnostic heterogeneity within psychosis. By reviewing relevant literatures, the authors aim to bridge studies showing connectome-wide associations within and between disorders with those establishing substantial between-person heterogeneity. They conclude with several practical recommendations and directions for the field. Overall this is a nice, concise and timely contribution that will help researchers think about where the field should go. I have a few comments that may improve the manuscript.

R1Q1: I appreciated the conceptual focus on different levels of phenotypic analysis, from individuals to diagnostic subgroups to diagnoses overall to transdiagnostic classes. This could be fleshed out more, however, as the connections between these layers seems to be the essence of the problem the authors want to solve. It was not always clear whether the authors think the proposed methodologies are uniquely suited to characterize one level of that hierarchy better than others, if one level is more important than others, or if they are simply intrinsically connected and should always be addressed simultaneously.

Response: We thank the reviewer for this insightful comment, which has prompted us to substantially strengthen the conceptual architecture of the manuscript. We fully agree that clarifying the hierarchical relationships between connectomic overlap and biotyping—and mapping appropriate methodologies to each level—is the core of this work's contribution.

To address this, we have restructured the core of the manuscript around a unified four-tiered inverted pyramid framework that systematically organizes transdiagnostic connectomic phenomena from macro-scale shared pathology down to micro-scale individual variation. The four tiers, their conceptual roles, and their corresponding methodological approaches are as follows:

1. Tier 1: Macro-scale transdiagnostic pathological scaffold

This top tier defines the broad shared pathological space of psychosis-spectrum conditions, constrained by whole-brain network architecture. Cross-disorder involvement mapping and connectome-wide association studies (CWAS) are best suited to characterize this level, as they identify recurrently disrupted hub regions, rich-club connections, and canonical large-scale systems that constitute a general transdiagnostic vulnerability scaffold.

2. Tier 2: Meso-scale phenotypic triangulation across diagnoses and behavior

This intermediate tier bridges biological circuits, traditional diagnostic categories, and dimensional behavioral phenotypes. Connectome-based predictive modeling (CPM) and cross-disorder phenotype association approaches are the primary methods here, used to test whether shared circuit

alterations underlie transdiagnostic symptom dimensions and whether disorder-specific circuit anomalies map to diagnosis-specific behavioral profiles.

3. Tier 3: Computational biotyping – data-driven neurobiological subgroups

This tier partitions the shared pathological space into discrete neurobiological subgroups based on distinct patterns of circuit deviation. Classical clustering algorithms, dimensionality-reduction enhanced clustering, and deep representation learning approaches are all employed at this level to identify reproducible biotypes that cut across diagnostic labels.

4. Tier 4: Interindividual variability

At the base of the pyramid lies person-specific connectomic deviation that cannot be fully captured by subgroup-level biotypes. Normative modeling is the core methodology for this tier, quantifying individualized deviations relative to a healthy reference population and enabling precision targeting at the individual patient level.

Crucially, these tiers are intrinsically connected and operate bidirectionally: the macro-scale scaffold constrains where pathological alterations can emerge across all lower tiers, while lower-tier heterogeneity in turn shapes the aggregate patterns observed at the macro level. Rather than positing one level as more important than others, the framework provides a hierarchical architecture that integrates shared vulnerability and heterogeneous pathology, and it maps specific analytical strategies to the scale at which they are most informative.

This full framework is elaborated in Section 2 of the revised manuscript and visually summarized in revised Figure 1.

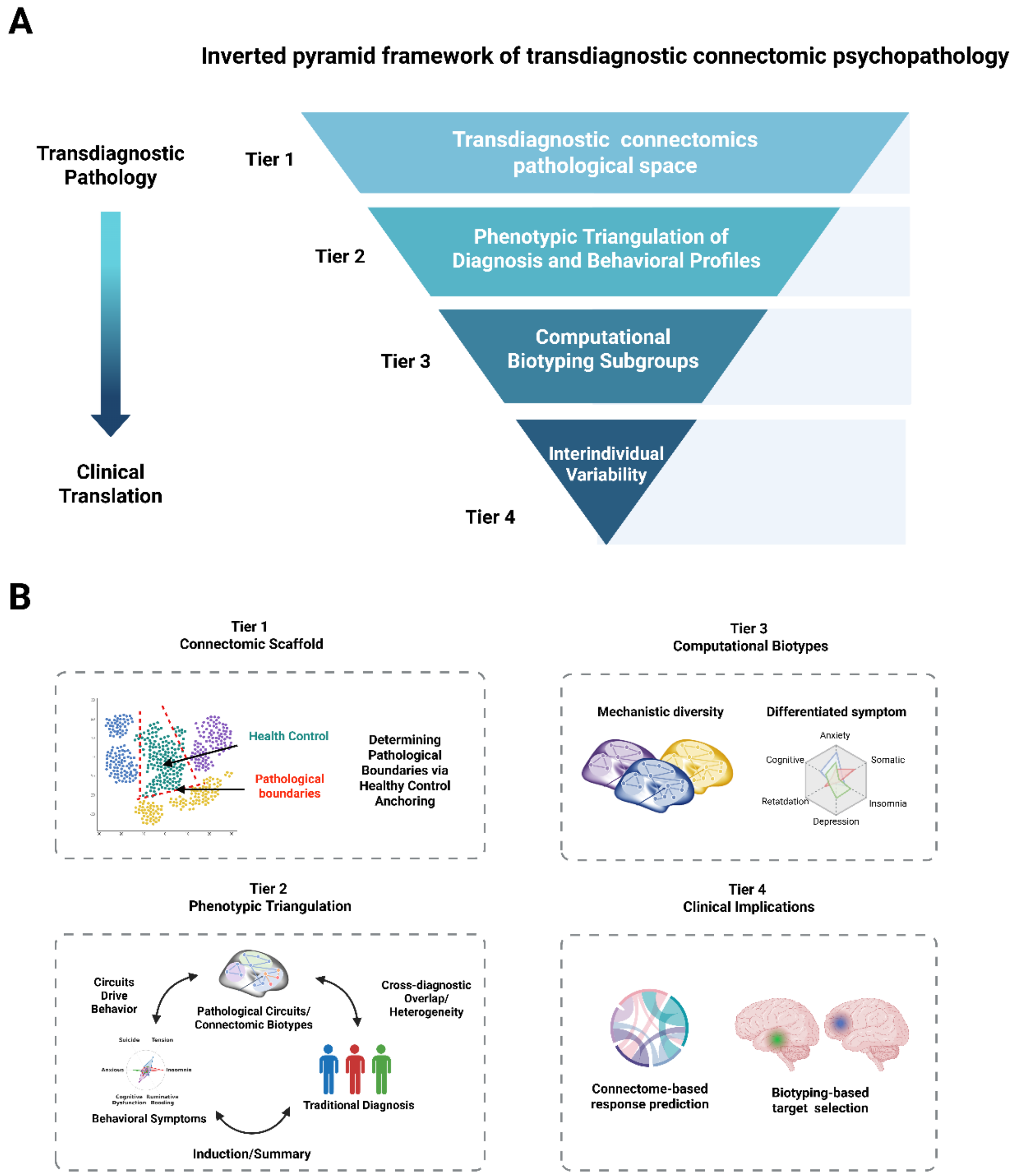

R1Q2: The authors note that the decisive criterion for validating biotypes is clinical utility. This is true, and the paper should provide a better description of where the field stands in terms of clinically useful connectomic signatures/biotypes/etc. Where are we now with actually using connectomics to inform treatment? How numerous, diverse, and strong are the studies? The authors note one individual study and one meta-analysis in line 274, but given that this is the primary objective of this work, stated a few times in the manuscript, context for where the field stands is important. This is especially the case given concerns that brain-level metrics, especially with available technology, may be insufficient for making real treatment decisions.

Response: We thank the reviewer for this important suggestion. We agree that contextualizing the current state of clinical translation is critical for framing our recommendations. We have therefore

added a comprehensive synthesis of the current evidence base and its limitations in the revised manuscript, both in Section 2.4 (Interindividual variability) and Section 3.3 (Moving Beyond Internal Metrics: Prospective External Validation of Biotypes).

The specific revisions in the manuscript are detailed below:

"Accumulating empirical evidence has begun to demonstrate the clinical utility of connectomic signatures for prognostic and predictive stratification across transdiagnostic psychiatric spectra. For psychosis spectrum conditions, Sarpal et al.50 established a resting-state fMRI–based striatal connectivity index that predicted antipsychotic response with an AUC = 0.78 in both first-episode schizophrenia and independent chronic psychosis cohorts, and further correlated with hospital length of stay. Extending beyond single-region seeds to whole-brain signatures, Cao et al.51 identified a paradigm-independent connectomic biomarker for individualized antipsychotic response in first-episode psychosis, with robust performance replicated in both discovery and independent validation samples. Beyond psychosis, Zhang et al.52 validated transdiagnostic generalizability in internalizing psychopathologies, showing that whole-brain functional connectivity predicted multidimensional symptom improvement across six diagnostic categories of depression and anxiety, and generalized across cognitive-behavioral therapy, pharmacotherapy, and supportive therapy modalities. Collectively, these findings span focal circuit markers to distributed connectome-wide signatures, cover both pharmacological and psychosocial interventions, and provide preliminary proof-of-concept for connectomics-guided clinical decision-making."

Updated references:

- Sarpal, D. K. et al. Baseline Striatal Functional Connectivity as a Predictor of Response to Antipsychotic Drug Treatment. Am. J. Psychiatry 173, 69–77 (2016).
- Cao, H. et al. A Functional Connectome-Based Neural Signature for Individualized Prediction of Antipsychotic Response in First-Episode Psychosis. Am. J. Psychiatry 180, 827–835 (2023).
- Zhang, K. et al. Functional Connectivity Predicting Transdiagnostic Treatment Outcomes in Internalizing Psychopathologies. JAMA Netw. Open 8, e2530008 (2025).

"Despite these promising findings, the clinical translation of connectomic biotyping remains at an early stage, constrained by notable evidence gaps and pervasive methodological limitations. Most available findings are derived from retrospective or single-site studies, and large-scale, multi-center prospective validation remains a critical barrier to routine clinical implementation. More broadly, Winter and Hahn53 have highlighted a systemic methodological flaw across the field: the majority of biotyping studies—including most deep learning–based connectomic subtyping work—fail to rigorously evaluate the statistical significance, stability, and out-of-sample generalizability of their derived subtypes. Instead, validity is frequently inferred solely from internal clustering performance, which is susceptible to arbitrary analytical choices and dataset-specific idiosyncrasies rather than reflecting clinically meaningful neurobiological heterogeneity.

To address this replication gap and strengthen clinical translatability, the evaluation of

connectomic biotypes must shift from a primary focus on internal clustering metrics to prospective, outcome-relevant external validation (see Fig. 2C). Divergent analytical pipelines—varying in feature definition, dimensionality reduction, distance metrics, and hyperparameters—can yield divergent "biotype" solutions even from the same dataset. For this reason, biotype validity cannot be judged primarily by within-cluster versus between-cluster indices or apparent stability under resampling. The decisive criterion is clinical utility: whether derived subtypes can prospectively predict clinical outcomes or differential treatment response beyond what is explained by conventional diagnostic labels and baseline symptom severity.

Two complementary, clinically oriented validation designs are particularly well suited to this goal. The first is pathology trajectory prediction, which forecasts the longitudinal evolution of symptoms and functional outcomes based on baseline connectomic profiles. In this paradigm, biotypes identified at study entry are tested for their ability to predict subsequent changes in symptom severity, illness course, and functional decline over months to years, independent of baseline diagnosis and clinical severity. This design directly assesses whether connectomic stratification captures prognostically meaningful neurobiological heterogeneity rather than transient cross-sectional differences, and can be implemented in naturalistic prospective cohorts without uniform treatment assignment. For example, Li et al.54 predicted baseline progression rate phenotypes in ALS from DTI white matter structural connectivity using linear classifiers, whereas Maheux et al.55 forecasted 1.5–4.5-year cognitive trajectories in Alzheimer's disease from multimodal baseline data, including hippocampal volumetry. The second is efficacy prediction, which examines whether baseline connectomic biotypes forecast differential response to specific interventions. Under standardized trial conditions, patients receive uniform treatments, and predictive validity is established by observing significantly divergent symptom improvement and connectomic remediation trajectories across subgroups. Early evidence in depression has demonstrated this principle by identifying connectivity-defined subtypes that predict responsiveness to repetitive transcranial magnetic stimulation56, and a recent large-scale study across multiple depression and anxiety cohorts confirmed that individualized circuit metrics can predict differential benefit from pharmacotherapy and behavioral therapy at scale57.

For transdiagnostic psychosis research, analogous validation efforts should prioritize prospective designs, harmonized intervention protocols, and preregistered endpoints. The goal is to demonstrate that connectome-derived subgroups generalize across sites and meaningfully stratify prognosis and treatment response. In this way, connectomic biotyping can advance from plausible substructure discovery to a clinically testable framework, and transdiagnostic connectomic overlap can be operationalized not only as a shared risk substrate but also as a set of constraints that guide robust, treatment-relevant stratification."

Updated references:

- Li, W. et al. Disruption of the white matter structural network and its correlation with baseline progression rate in patients with sporadic amyotrophic lateral sclerosis. Transl. Neurodegener. 10, 35 (2021).
- Maheux, E. et al. Forecasting individual progression trajectories in Alzheimer's disease. Nat. Commun. 14, 761 (2023).

- Tozzi, L. et al. Personalized brain circuit scores identify clinically distinct biotypes in depression and anxiety. Nat. Med. 30, 2076–2087 (2024).

The recommendations are strong and appreciated.

Response: We greatly appreciate the reviewer’s positive appraisal of our recommendations. We are glad they were deemed constructive. In the revised manuscript, aligned with overall content adjustments, we have further strengthened the operational feasibility and supporting rationale for these recommendations.

Responses to reviewers

Reviewer: 2

Dear authors, thanks for sharing with us this manuscript. Here is my review.

R2Q1: The central problem with this manuscript is that its contribution relative to the existing literature is unclear. The transdiagnostic connectomics perspective has been articulated in prior work, including Buckholtz and Meyer-Lindenberg (2012), which the authors cite as reference [18], and Vanes and Dolan (2021), cited as reference [19]. The conceptual distinction between shared network-level constraints (overlapping) and individual deviation patterns (biotyping) is a useful organizational device, but it does not constitute a novel theoretical advance.

Response: We appreciate the reviewer's perspective and fully agree that explicitly delineating our conceptual contribution relative to prior foundational work is essential. We have revised the manuscript to clarify that our core innovation lies not in proposing transdiagnostic connectomics itself, nor in separately describing overlap or biotyping, but in integrating these two previously parallel lines of inquiry into a mechanistically progressive, four-tiered hierarchical framework that specifies their bidirectional functional relationship.

Earlier seminal work (Buckholtz & Meyer-Lindenberg, 2012; Vanes & Dolan, 2021) established the connectome as a transdiagnostic substrate of psychiatric risk and catalogued shared cross-disorder neural alterations. Subsequent biotyping work has focused on subgroup identification, largely separate from the transdiagnostic overlap literature. What has been missing is a unifying architecture that explains how macro-scale shared network constraints and micro-to-meso scale heterogeneous deviation patterns mechanistically interact to produce the clinical landscape of psychosis.

Our inverted pyramid framework fills this gap by organizing transdiagnostic connectomics into four hierarchically nested tiers:

1. Macro-scale transdiagnostic pathological scaffold – the shared network vulnerability architecture
2. Meso-scale phenotypic triangulation – the mapping of circuits to diagnoses and behavioral dimensions
3. Computational biotyping – data-driven neurobiological subgroups defined by distinct deviation patterns
4. Interindividual variability – residual person-specific pathology beyond subgroup-level patterns

Within this framework, connectomic overlap is not merely a descriptive observation; it acts as a "common scaffold" that constrains where pathological alterations can emerge across all disorders and individuals. Biotyping, in turn, partitions the residual variance around this scaffold into pathophysiologically distinct subgroups. Critically, the relationship is bidirectional: overlap-informed priors can stabilize biotyping solutions and reduce spurious clustering driven by sampling or site effects, while reproducible biotypes can clarify whether apparent overlap is truly universal

or dominated by specific subgroups. This integrated, hierarchical account of transdiagnostic heterogeneity represents the primary conceptual advance of our work, and it is elaborated in full in Section 2 of the revised manuscript.

R2Q2: The recommendations in the final section, namely standardized data acquisition, normative anchoring, and prospective validation, reflect established best practices in the field rather than new proposals.

Response: We agree with the reviewer that these three principles represent broadly recognized best practices in neuroimaging research. Our contribution lies not in inventing these concepts, but in systematically tailoring and operationalizing them specifically for the transdiagnostic connectomic biotyping of psychosis—a domain where implementation remains fragmented and consensus on practical execution is lacking.

Rather than restating general principles, we provide concrete, field-specific implementation roadmaps tied to emerging state-of-the-art practices:

- For data integration and harmonization, we contrast two complementary, actionable strategies: (1) standardized "HCP-like" prospective acquisition exemplified by the Transdiagnostic Connectome Project (TCP), which prioritizes measurement precision and cross-site reproducibility; and (2) legacy multi-cohort reuse with harmonized processing exemplified by Reproducible Brain Charts (RBC), which enables rapid scaling and broad diagnostic coverage. We explicitly outline the tradeoffs, ideal use cases, and best practices (such as BIDS-format open data release) for each strategy.
- For normative anchoring, we move beyond general endorsement of normative modeling to specify how healthy-anchored deviation profiles should be integrated into biotyping workflows. We highlight the HYDRA framework as a paradigmatic example of end-to-end anchor-based subtyping, and we argue that clustering deviation profiles (rather than raw connectomes) is critical for avoiding nuisance-driven clusters. We also introduce contrastive learning approaches (e.g., contrastive PCA) as a complementary technique for isolating disease-specific signals from background noise.
- For prospective validation, we define two distinct, clinically meaningful validation paradigms—pathology trajectory prediction and treatment efficacy prediction—and outline how each should be operationalized for transdiagnostic psychosis research. We explicitly argue for moving beyond internal clustering metrics as the primary validity criterion, a shift that remains insufficiently implemented in the current biotyping literature.

These targeted, operationalized recommendations are grounded in the latest emerging methodologies and are tailored to the specific challenges of transdiagnostic psychosis connectomics. This section is presented in full in Section 3 of the revised manuscript.

R2Q3: The scope of the literature covered is also relatively narrow for a review paper. A narrative, non-systematic and non-quantitative review can be valuable when it synthesizes a broad literature

into a new interpretive framework or when it identifies non-obvious gaps, but the present manuscript does not attempt comprehensive coverage, does not perform any quantitative synthesis, and does not propose a genuinely novel conceptual model. I would encourage the authors to consider what specific question or gap motivated this review and to restructure the manuscript around that contribution, whether it takes the form of a systematic synthesis, a novel theoretical framework, or an original analysis. The topic itself is timely and clinically important, and there is room for a review that makes a distinctive contribution to the transdiagnostic connectomics literature.

Response: We thank the reviewer for this important feedback, which has prompted us to clarify the positioning and scope of the manuscript. First, we wish to confirm that this work is framed as a Perspective article rather than a systematic or comprehensive review. Accordingly, its goal is not to exhaustively survey the literature or perform quantitative synthesis, but to put forward a novel integrative conceptual framework and a forward-looking translational roadmap for the field. We have explicitly stated this positioning in the revised Introduction.

The core gap that motivated this work is the persistent disconnection between two largely separate research streams: the literature on transdiagnostic shared connectomic alterations, and the literature on data-driven biotyping of psychiatric disorders. These two bodies of work have advanced largely in parallel, with little integration of how shared network constraints and heterogeneous deviation patterns mechanistically interact. Our primary contribution is addressing this gap by proposing the four-tiered inverted pyramid framework, which unifies these two streams into a single hierarchical architecture and explicates their bidirectional functional relationship.

Grounded in this framework, the manuscript further makes three distinctive contributions:

1. It articulates a mechanistic account of how connectome architecture shapes both shared vulnerability and heterogeneous pathology across diagnostic boundaries.
2. It provides operationalized, field-specific implementation guidelines for data harmonization, healthy-anchored biotyping, and prospective clinical validation, tied to the latest emerging methodologies.
3. It outlines a concrete translational roadmap for moving connectomic biotyping from descriptive subgroup discovery to clinically actionable stratification in precision psychiatry.

We have restructured the manuscript around this core contribution, with Section 2 dedicated to the inverted pyramid framework and Section 3 dedicated to the translational roadmap and recommendations. We believe this structure aligns with the standards for a Perspective article and delivers a distinctive, forward-looking contribution to the transdiagnostic connectomics literature.
Again, we thank the reviewer for their thoughtful feedback, which has been instrumental in helping us clarify and strengthen the contribution of this work.

Responses to reviewers

We sincerely thank both reviewers for their thoughtful and constructive comments, which have greatly helped us refine and strengthen the manuscript. Our detailed responses to each comment are provided below.

Reviewer: 1

The authors have done a nice job of addressing my prior comments. I think the paper is improved.

R1Q1: The new figures are especially interesting. I do have one constructive comment about the inverted pyramid framework though. It seems to combine biological/psychopathological levels of observation with the methodologies used to identify them. For the figure to be most helpful, these should probably be separated if both are intended to be included. At a minimum, it should be explicitly acknowledged that structures and methods are reflected, and probably each level of the hierarchy should display both levels of observation and methods (if both are deemed equally relevant by the authors).

Response: We thank the reviewer for the constructive feedback, which has greatly helped enhance the framework of our manuscript. We fully agree with the need to clarify the pathological observations and analytical methodologies to reflect their equal importance in this study.

To this end, we have restructured Figure 1 to highlight the observation of pathological phenomena in Figure 1A, while emphasizing the corresponding methodology for each layer in Figure 1B. Specifically, we modified the subheadings for each layer in Figure 1B to underscore their methodological focus. Furthermore, we revised the title of the fourth layer from "Interindividual Variability" to "Biotype-guided Translation" to emphasize its primary core in biotype-guided clinical translation. In line with this focus, we also incorporated longitudinal prediction and external data validation into the Clinical Translation section of Figure 1B.

For specific textual and visual modifications, please refer to the revised manuscript and the updated Figure 1.

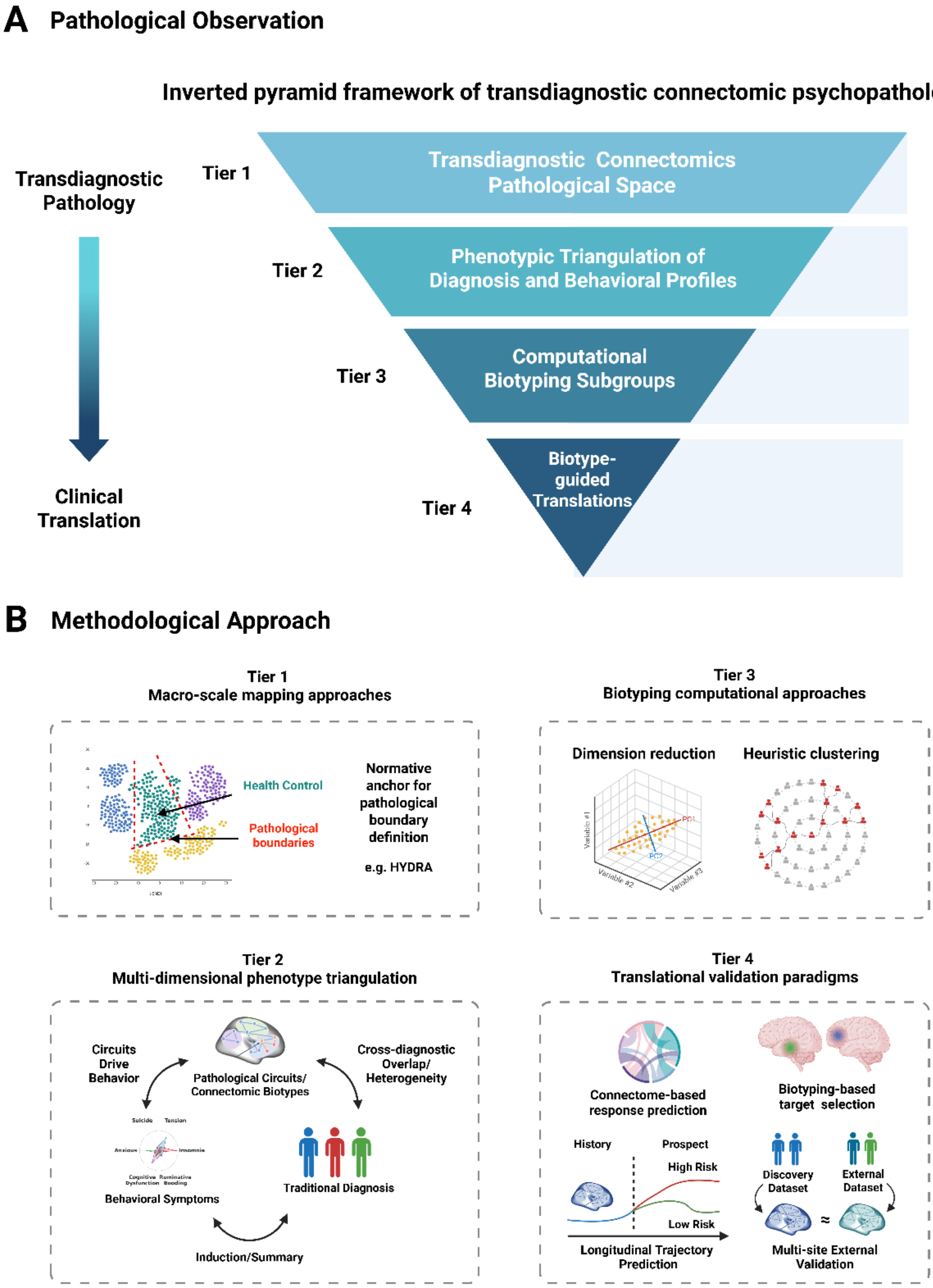
A
Pathological Observation
Inverted pyramid framework of transdiagnostic connectomic psychopathology
Transdiagnostic Pathology
Clinical Translation
Tier 1
Transdiagnostic Connectomics Pathological Space
Tier 2
Phenotypic Triangulation of Diagnosis and Behavioral Profiles
Tier 3
Computational Biotyping Subgroups
Tier 4
Biotype-guided Translations
B
Methodological Approach
Tier 1
Macro-scale mapping approaches
Health Control
Pathological boundaries
Normative anchor for pathological boundary definition
e.g. HYDRA
Tier 3
Biotyping computational approaches
Dimension reduction
Heuristic clustering
Tier 2
Multi-dimensional phenotype triangulation
Circuits Drive Behavior
Pathological Circuits/ Connectomic Biotypes
Cross-diagnostic Overlap/ Heterogeneity
Behavioral Symptoms
Traditional Diagnosis
Induction/Summary
Tier 4
Translational validation paradigms
Connectome-based response prediction
Biotyping-based target selection
History
Prospect
High Risk
Low Risk
Longitudinal Trajectory Prediction
Discovery Dataset
External Dataset
Multi-site External Validation

Reviewer: 2

Dear authors,

thanks for addressing my (our) revisions. I don't have any further objection towards publication.

Response: We thank the reviewer very much for your time and positive feedback. We sincerely appreciate your constructive comments during the revision process, which have greatly helped improve the quality of our manuscript.
